# Sustainability and Polyesters: Beyond Metals and Monomers to Function and Fate

**DOI:** 10.1021/acs.accounts.2c00134

**Published:** 2022-05-17

**Authors:** Guilhem X. De Hoe, Theona Şucu, Michael P. Shaver

**Affiliations:** † Department of Materials, School of Natural Sciences, University of Manchester, Manchester M1 3BB, United Kingdom; ‡ Henry Royce Institute, University of Manchester, Sustainable Materials Innovation Hub, Manchester M13 9BL, United Kingdom

## Abstract

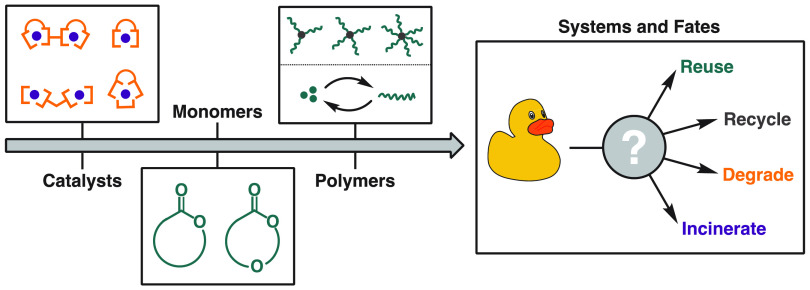

Poor waste management and unchecked consumption underpin our current paradigm of plastics use, which is demonstrably unsustainable in the long term. Nonetheless, the utility and versatility of plastics suggest that the notion of a plastic-free society is also unsustainable. Responses to this conundrum are increasing, and among these are research efforts focused on the development of more sustainable plastics. This Account, written by trained chemists, reflects an academic research journey culminating in an appreciation of the importance of improving and enabling the overarching systems that plastics exist within. Our primary initial focus was on catalyst development because catalysts are key drivers of sustainability by improving the efficiency and ease of polymerization. Metal catalysts ranging in ligand structure and the incorporated metal(s) were developed for the preparation of traditional polyesters such as poly(lactic acid) and polycaprolactone. The central themes in these works were stereocontrol (tacticity), efficiency (polymerization rate), and versatility (monomer scope). Alongside insights gained by systematically varying catalyst structure came impressive results gained through collaboration, including the remarkably high activity of novel heterometallic zinc catalysts toward various cyclic esters.

This catalysis work was complemented by and slowly transitioned to a focus on polymer functionality and monomer design. Several fundamental studies focus on polymer topology, specifically star-shaped polyesters, tuned arm number, length, and tacticity. These reports feature emphases on the end of life (solvolysis) and physical properties of polymers, which were increasingly important themes as work shifted toward new methods of incorporating functionality in polymers produced by ring-opening polymerization. Three key highlights demonstrate this shift: the first two rely upon the exploitation of olefin metathesis (cross- and ring-closing) to functionalize polyesters or polyethers, and the third involves the manipulation of ring-opening polymerization equilibrium to enable selective monomer recovery from a polyester. Our foundational work on 1,3-dioxolan-4-one (DOX) monomers is then discussed because this emerging class of molecules offers a distinct synthetic pathway toward functional polyesters, both conventional and novel. With this DOX framework, polyesters that are usually challenging to synthesize (e.g., poly(mandelic acid)) are accessible because polymerization is driven by the concomitant, controlled extrusion of small molecules (acetone or formaldehyde).

After these polyester-focused highlights, the foundation of our ongoing work is presented, namely, that polymer sustainability must be viewed from a systems-level perspective, including economic and social components alongside the environmental considerations. Material design must be driven by practice, and we have to involve key players in academia, industry, and government in a concerted effort to enable positive and robust change. The key goal is to develop sustainable systems that retain plastics in their highest value state for as long as possible by designing materials and products for a particular (and assured) end-of-life fate, whether that be reuse, recycling, (bio)degradation, or energy recovery.

## Key References

- GruszkaW.; LykkebergA.; NicholG. S.; ShaverM. P.; BuchardA.; GardenJ. A.Combining Alkali Metals and Zinc to Harness Heterometallic Cooperativity in Cyclic Ester Ring-Opening Polymerisation. Chem. Sci.2020, 11, 11785–1179010.1039/D0SC04705H34123205PMC8162475.^1^*Two heterometallic Na/Zn*_2_*and K/Zn*_2_*catalysts were reported, and their outstanding activity was illustrated in the (co)polymerizations of cyclic esters*.
- CairnsS. A.; SchultheissA.; ShaverM. P.A Broad Scope of Aliphatic Polyesters Prepared by Elimination of Small Molecules from Sustainable 1,3-Dioxolan-4-Ones. Polym. Chem.2017, 8, 2990–299610.1039/C7PY00254H.^2^*An array of structurally diverse polyesters such as poly(mandelic acid) is produced from easy-to-synthesize 1,3-dioxolan-4-ones*.
- BurgessM.; HolmesH.; SharminaM.; ShaverM. P.The Future of UK Plastics Recycling: One Bin to Rule Them All. Resour. Conserv. Recycl.2021, 164, 10519110.1016/j.resconrec.2020.105191.^3^*Contextualization of multiple fates for polymers viewed through polymer properties, government policy, economic cooperation, and behavioral understanding*.

## Background and Context

There is arguably no such thing as a sustainable plastic.^4^ We could consider the source, where a push to bioderived monomers decouples industry from its dependence on petroleum, or recycling, where improved mechanical and chemical recycling can enable the circular economy, or (bio)degradation, where decomposition to useful chemicals (e.g., monomers) or benign molecules (e.g., CO_2_ and H_2_O) eliminates residual harm.^5−8^ Each of these could be sustainable if enabled by a system that ensures the imagined fate is realized. Each of them could not be sustainable if this valuable resource continues to be mismanaged or if we forget that our plastic usage is intimately interrelated to our carbon footprint. Our global plastics crisis is a crisis because of unmanaged waste and unfettered consumption, not performance.

The three authors of this Account were originally trained as chemists. This central science is key to the creation of our plastic addiction but also essential to unlocking a sustainable future. We must ensure, however, that we avoid unintended consequences in this quest. Our linear plastics economy was created through technocentric solutions that exacerbate global sustainability as they market the solution instead of exploring the unintended consequences.^9^ This perspective thus represents a journey in changing perspectives, from an original focus on catalyst design to improving the process of making polymers, focusing on topology and monomer choice, changing the fate of the polymers themselves, and finally realizing that innovation may fail if it does not incorporate social and economic sustainability into environmental sustainability perspectives.

Within this context, this Account will focus on polyesters. From specialist biomedical polymers to ubiquitous commodity packaging, these backbones have touched every aspect of our lives.^10−16^ They also are a showcase for why it is important to consider the entire lifecycle because there are multiple potential fates to consider, some established and some emerging. Polyesters can be circularized through mechanical recycling (recovering materials for reuse through physical means such as grinding, washing, separating, and reprocessing), chemical recycling (either nonselective pyrolysis or through selective depolymerization/solvolysis to monomers^17^), biodegradation (breakdown from the action of naturally occurring microorganisms), or composting (biodegradation under controlled conditions).

As polymer chemists, we must ensure that design needs to be informed by practice, not practice changed by design. We must ensure that the polymers we create have an assured fate: it does not matter if a polymer is recyclable or biodegradable or compostable if it is not actually recycled, biodegraded, or composted. This focus on systems and past tense terminology highlights that there are no panaceas. A truly sustainable system is one which incorporates multiple ends of life.

Understanding the importance and interrelationship of these fates has been a journey. This Account will begin with the siloed perspective of an inorganic chemist, with the aim of developing improved catalysts for existing systems. It will transform into an understanding that accessible properties do not meet real world demands and an expansion of function and topology is needed to achieve the expected performance. It ends where we now begin: an understanding that our imagined fates must be assured, leading to our current interdisciplinary team of chemists, polymer scientists, and social scientists aiming to develop not just polymers but the system which enables their sustainability.

## Catalysis

Given its critical role in reaction rates, energy usage, and selectivity, catalysis remains an essential facet of sustainability in both molecular and macromolecular synthesis.^18,19^ Cleaner, faster, flexible systems drive innovation in sustainable polymers, and metal-based catalysts have historically dominated this field.^7,20,21^ In aliphatic polyesters, the catalytic ring-opening polymerization (ROP) of cyclic esters offers high yields, mild polymerization conditions, and unparalleled control over the polymer molecular weight (*M*_n_), dispersity (*Đ*), end group, and architecture.

**Figure 1:**
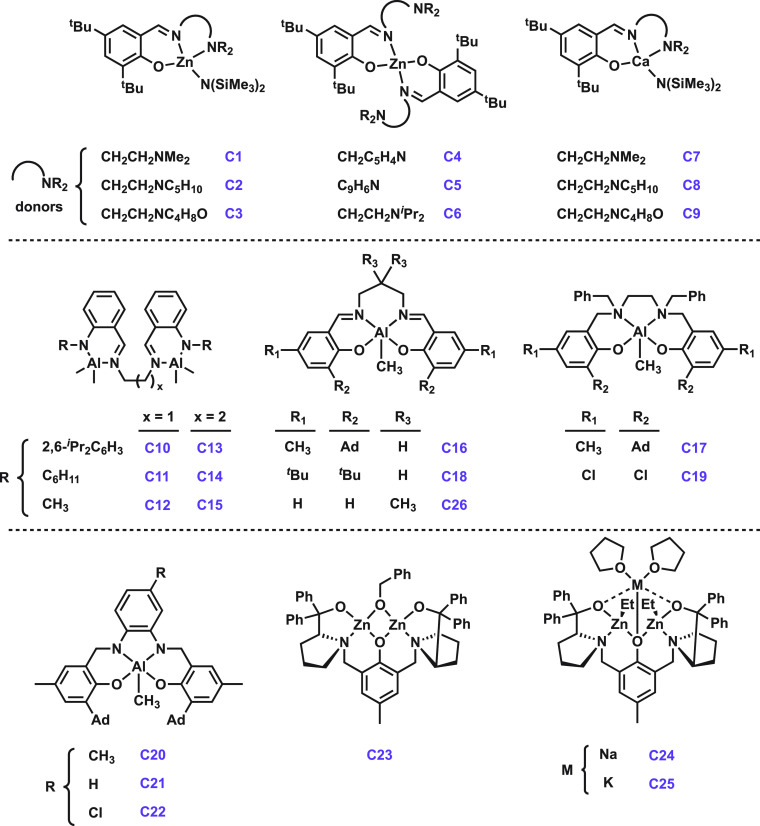
Catalyst scope for the ring-opening polymerization of lactones and other cyclic monomers.

Catalyst design was our first pathway into sustainable polymer synthesis, both through independent study and, increasingly, through collaboration. It provided us not only interesting results but also new perspectives on the successes and failures of ligand design and polymer properties informing future efforts in monomer design. Some time ago, we designed zinc and calcium complexes supported by phenoxyimine ligands (**C1**–**C9**) with various pendant donors for traditional aliphatic polyesters such as poly(lactic acid) (PLA) and polycaprolactone (PCL) (Figure 1). Controlling the ligand coordination sphere was essential, with ligands moderating the Lewis acidity, steric environment, and accessible initiating sites. Although **C1**–**C3** readily facilitate productive polymerization, disproportionation leading to bis-ligated complexes is commonplace and can shut down polymerization (**C4**–**C6**) or promote insertion into metal–ligand bonds, where poor initiation disconnects [M]/[I] ratios from the observed molecular weight. This observed rearrangement is, of course, metal-dependent, with calcium complexes (**C7**–**C9**) avoiding disproportionation albeit with lower reactivity.^22^

This catalyst-led approach can also be exploited to control tacticity, as is now commonplace in lactide polymerization.^23,24^ We envisaged salen ligands where the bulky substituents were moved from the ortho position to the amido donor of an anilido-aldimine framework. In catalysis, bulk is clearly a balancing act because this proximity precluded the formation of monometallic complexes, although the bimetallic dimethyl aluminum complexes (**C10**–**C15**) remained active. This work provided another example of ligand noninnocence, where **C10** promoted uncontrolled polymerization with a multimodal molecular weight distribution attributed to insertion into pendant ligand arms. The goal of improved tacticity control was also misguided because the opening of the coordination sphere led to atactic polymers.^25^ To properly enhance tacticity control, novel monometallic complexes with methyl and adamantyl substituents on the phenolate rings were designed (**C16** and **C17**). **C16** was able to mediate the ROP of *rac*-lactide, with the resulting PLA showing an 88% isotactic enchainment, contrasted with **C17** where the bulk now prevented productive polymerization.^26^

Although we retained good to excellent catalyst yields (50–80%) and were continuing to improve their performance, all of these efforts felt quite iterative: slightly faster, slightly higher, and slightly better. We thus turned our focus to other cyclic esters. Copolymerizations of *rac*-lactide and *rac*-β-butyrolactone with known commercial or established catalysts (Sn(Oct)_2_ or **C18** and **C16**) afford relatively uncontrolled, atactic poly(lactic acid)-*co*-poly(3-hydroxybutyrate) (*Đ* > 1.5). Al-salan complex **C19** provided unprecedented control over the molar mass distribution (*Đ* < 1.09), irrespective of monomer feed ratios. Kinetic studies confirmed that the insertion rate of *rac*-lactide was faster than for *rac*-β-butyrolactone, forming long heterotactic PLA segments within the copolymers, although DSC analysis suggested no phase separation between the two polyester segments.^26^ Tuning electronics through switching to phenyl bridges in **C20**–**C22** improved the lactide polymerization rates, but the resulting PLA was atactic, attributed to the rigid phenylene backbone minimizing steric clashes with the growing polymer chain and incoming monomer. **C20**–**C22** afforded poly(3-hydroxybutyrate) with syndiotactic microstructures and again consistently increased the polymerization rates.^27^

We investigated the effect of varying β-lactone substituents (methyl, ethyl, and *n*-butyl) on polymerizations catalyzed by **C18**. Through sequential monomer addition, we successfully obtained ABA triblock copolymers with poly(l-lactide) end blocks and poly(*rac*-β-butyrolactone), poly(β-valerolactone), or poly(β-heptanolactone) midblocks. Copolymers synthesized from *rac*-β-butyrolactone or β-valerolactone yielded materials with tunable thermal properties (*T*_g_ varied between −20 and 60 °C), whereas using β-heptanolactone as a comonomer led to microphase-separated morphologies, as evidenced by two distinct *T*_g_ values and small-angle X-ray scattering results.^28^

The versatility of salen- and salan-based complexes extends to seven-membered aliphatic cyclic esters as well. We found that **C18** gave exceptionally high levels of control as well as the fastest polymerization rates for ε-caprolactone, even though previous reports suggested Al-mediated caprolactone polymerizations were not feasible.^29^ PCL with high molecular weight and narrow dispersity (*M*_n_ = 175 kg mol^–1^ and *Đ* = 1.04) was achievable at room temperature in just 6 h. High conversions (≥94%) were also observed when **C18** was used to polymerize δ-valerolactone, although with slightly increased dispersity (*Đ* = 1.16). On the other hand, **C19** enabled similar conversions (>90%) without compromising dispersity (*Đ* < 1.1). Using optimized conditions, we studied the polymerization of two substituted monomers, 6-methyl-ε-caprolactone and 2,6-dimethyl-ε-caprolactone. Because of steric interactions between the lactone substituents and the active catalyst metal center, the reaction temperature had to be increased to facilitate the ROP of 6-methyl-ε-caprolactone. No polymerization was observed for 2,6-dimethyl-ε-caprolactone, even at these higher temperatures, again showcasing the importance of this steric balancing act.^29^

By this stage, we were realizing that our interests and strengths are in the polymers themselves. However, we continue to collaborate in this space, providing the polymer perspective for inorganic chemists. This is particularly true for our growing body of work with Jennifer Garden at the University of Edinburgh, who continues to highlight the importance of bimetallic complexes in ROP catalysis. With her team, we exploited metal–metal cooperativity in **C23**, a dinuclear zinc catalyst using a Trost ProPhenol ligand. Polymerizations of ε-caprolactone and *rac*-lactide using **C23** were remarkably fast, yielding a block polymer with narrow dispersity (*Đ* = 1.1) in just 7 min. Another unprecedented result using **C23** was the ability to prepare poly(ε-caprolactone-*block*-lactic acid-*block*-β-butyrolactone) in a one-pot reaction with excellent control (*Đ* = 1.13).^30^ Combining the high activities of Na and K with good control provided by Zn, we also reported **C24** and **C25** which outperformed homometallic catalysts for the ROP of ε-caprolactone, *rac*-lactide, and δ-valerolactone. **C25** proves to be the fastest heterometallic catalyst for *rac*-lactide ROP, affording PLA with *Đ* = 1.40. **C24** produces PCL with *M*_n_ = 19.6 kg mol^–1^ in only 4 min under ambient conditions, whereas **C25** proved to be less reactive likely because the larger, more electropositive metal center in **C24** promotes the coordination of ε-caprolactone.^1,31^ These results demonstrated the potential for heterometallic catalysts to provide unprecedented ring-opening polymerization rates. Although the exact mechanism for these rate enhancements is not yet clear, we suspect that the interplay between the Lewis acidity of the electropositive metal (e.g., Na or K) and the strength of the M–OR bonds around the electrophilic metal (Zn) can be fine-tuned to optimize performance.

## Topology

As we were developing our understanding of a broader monomer set through catalysis, it became clear that the polymer properties themselves would leave much to be desired. Polymer properties such as solubility, melt viscosity, thermal properties, and physical processing can be altered through topology control.^32^ Star polymers, characterized by multiple polymer “arms” radiating from a central core, have sparked significant interest within multiple fields because of the increased number of chain-end functionalities and enhanced control over degradation time scales as compared to linear polymers.^33−35^ We explored structure–property relationships for this class of polymers to better inform future material design.

**Figure 2:**
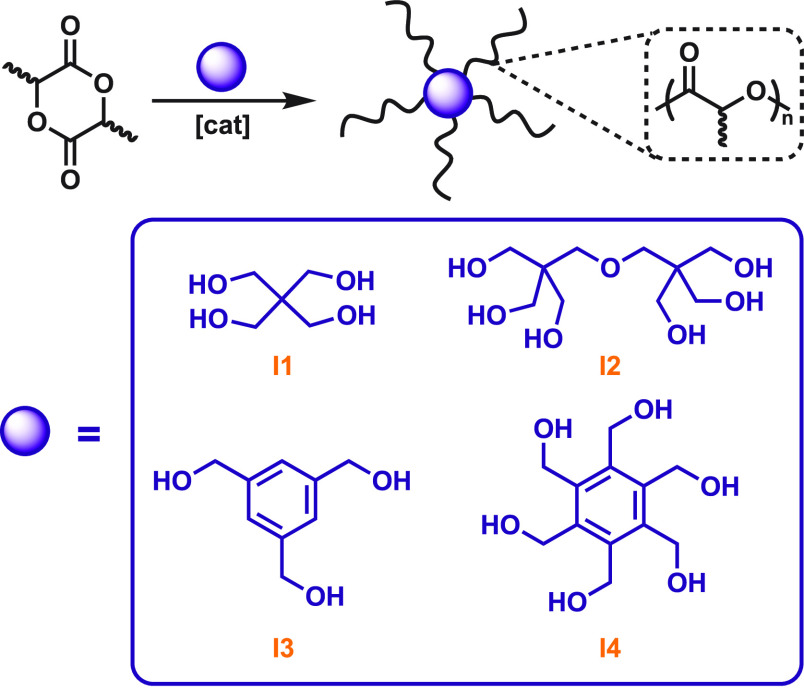
Cores investigated for topology-related studies of star-shaped poly(*rac-*lactide).

Early on, we wanted to show that the physical properties of polymer stars depend more on the individual arm length than the absolute molecular weight. We designed a series of star polyesters from *rac*- and l-lactide and flexible (**I1**, **I2**) or rigid arene (**I3**, **I4**) polyol initiators (Figure 2). For a given molecular weight, *T*_g_ was independent of the core structure yet dependent on the number of arms. However, melting temperatures between six-armed isotactic PLA stars of similar molecular weight did change; for example, the **I4**-based star had a *T*_m_ that was 20 °C higher than that of **I2**-based polymers, suggesting an influence from the core rigidity.^34^ We also targeted dendritic tetraols containing aliphatic or aromatic backbones: we found that *T*_g_ and *T*_m_ values are reliant upon the individual arm length, whereas the total molecular weight of the star has an additional effect on crystallization.^35^

The marriage of topology and tacticity is also of merit. Six-armed polymer stars built on a core molecule of **I2** can be prepared using Sn(Oct)_2_ and aluminum catalysts (**C18**, **C19**), affording polymer arms with atactic, heterotactic, and isotactic biases from *rac*-lactide or l-lactide. The systems maintained the tacticity control displayed in linear PLA synthesis, improving degradation temperatures by over 50 °C with topology^36^ while tuning *T*_g_ and *T*_m_ through microstructure control.^37^ Solvolysis with methanol in the presence of 1,5,7-triazabicyclo[4.4.0]dec-5-ene (TBD) showed that this tuning extended to degradation, as atactic and heterotactic stars degraded quickly (∼20 min) relative to isotactic PLA stars (∼3 h). This work additionally highlighted the effect of stereoerrors on sample stability.^36,37^

## Functionality

**Figure 3:**
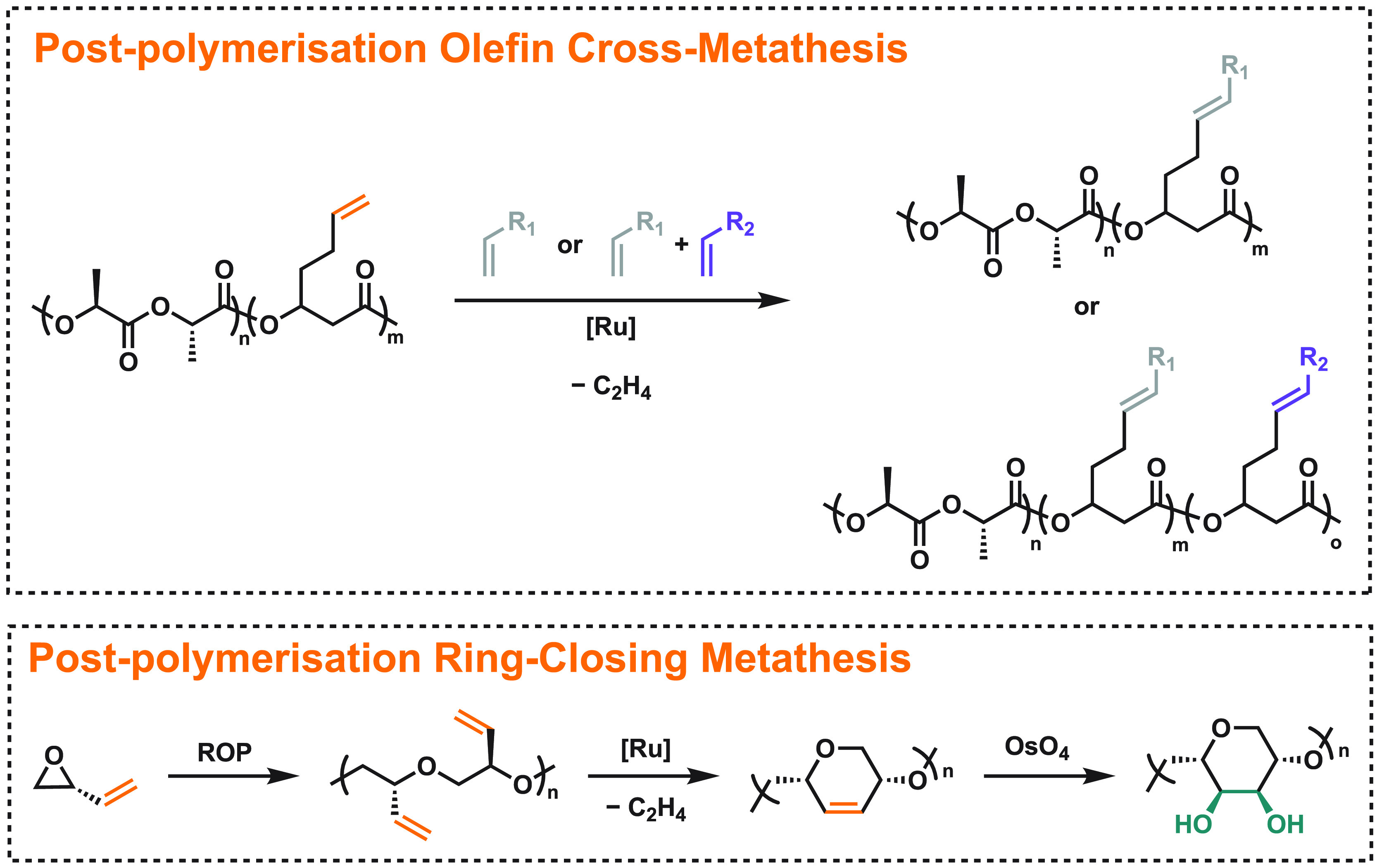
Olefin metathesis as an effective tool for functionalizing polyesters (through cross-metathesis, top) and polyethers (through ring-closing metathesis, bottom).^40,41^

In exploring the solvolysis of traditional aliphatic polyester systems, we were transitioning from an inorganic catalysis group to a team with a polymer chemistry focus. If we could not achieve the properties we wanted from commercially available monomers, did we need to design our own? Could we change the function or fate of the polymers? We identified olefin metathesis as a promising approach to novel, functionalized aliphatic polyesters and polyethers (Figure 3).^38^ We first investigated a novel exocyclic olefin derivative, (3*S*,6*S*)-3,6-dimethyl-5-methylene-1,4-dioxan-2-one. Prepolymerization functionalization through cross-metathesis with methyl acrylate and hex-1-ene afforded thermally unstable products, so we focused our efforts on a 3-methylenated lactide, (6*S*)-3-methylene-6-methyl-1,4-dioxan-2,5-dione. This monomer undergoes alcoholysis rather than productive ROP, and we hypothesized that cross-metathesis with hex-1-ene followed by hydrogenation with Pd/C would lead to a more stable monomer. This was indeed the case, and Sn(Oct)_2_-catalyzed ROP yielded a polyester with *M*_n_ = 9.7 kg mol^–1^, *Đ* = 1.3, and *T*_g_ = 1 °C.^39^ Prepolymerization functionalization of β-heptenolactone was also investigated. Metathesis with methyl acrylate proved successful, but subsequent monomer purification was cumbersome.

The most productive pathway was through postpolymerization functionalization (Figure 3). Homo- and copolymers derived from β-heptenolactone could be readily functionalized with methyl acrylate and 1,2-epoxy-5-hexene with >90% incorporation. Incorporating these new functional groups modified the thermal transitions (*T*_g_ by up to 40 °C) and stability (*T*_d,5%_ by up to 50 °C). To target a more economically sustainable product, this strategy was applied to copolymers of l-lactide and β-heptenolactone that could be further functionalized via metathesis. A much broader substrate scope was opened up, providing the easy introduction of aliphatic, aromatic, acrylate, epoxy, silane, and phosphonate groups to a single starting polymer.^40^

In studying this system, we recognized that ring-closing was a competing reaction pathway in β-heptenolactone homopolymers. In an effort to avoid this fate, we wondered if it could also facilitate access to synthetic polysaccharide mimics. 1,4-Linked six-membered cyclopolyethers could be generated via the ring-closing olefin metathesis of polyepoxybutene (Figure 3). After optimizing the reaction conditions with atactic polyepoxybutene, we prepared isotactic polyepoxybutene (*M*_n_ = 4 kg mol^–1^, *Đ* = 1.15) and quantitatively converted the pendant alkenes to cyclopolyether units. Subsequent diastereoselective dihydroxylation yielded a new stereodefined polymer, which possessed a hydrophilic surface due to the cis diols. This functional poly(cyclopolyether) is similar in structure to amylose, with a slightly less rigid backbone due to the extra methylene in the repeat unit structure.^41^

**Figure 4:**
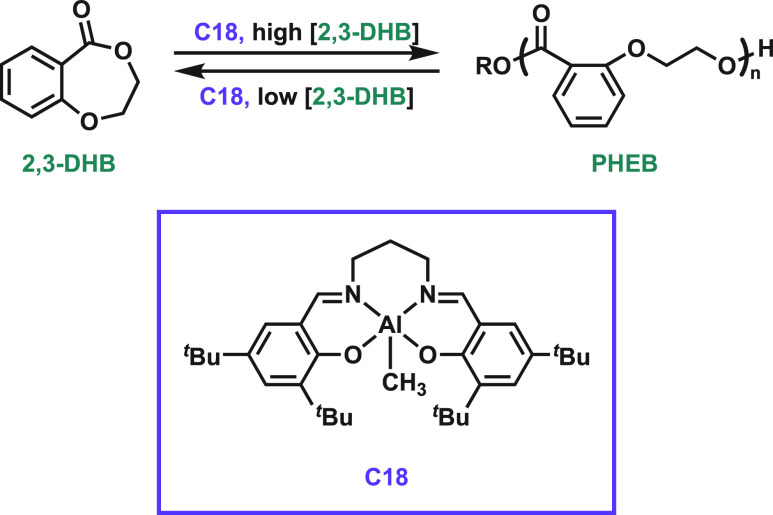
Catalytic (de)polymerization of poly(2-(2-hydroxyethoxy)benzoate) (PHEB): ring-opening polymerization at high 2,3-DHB concentrations and ring-closing depolymerization at low 2,3-DHB concentrations.

Of course, none of these structures address the underlying sustainability of the polymers themselves. Does it matter if polymers degrade if we have expended rare and/or toxic metals in their production? We were interested in the potential for ring structures to promote circularity and thus targeted the ROP of 2,3-dihydro-5*H*-1,4-benzodioxepin-5-one (2,3-DHB, Figure 4).^42,43^**C18**-catalyzed polymerization generated poly(2-(2-hydroxyethoxy)benzoate) (PHEB) with *M*_n_ values of as high as 80 kg mol^–1^ at high monomer conversions. This system exhibits concentration-dependent circularity through reversible cycles from high (4.1 M, 82% conversion to PHEB) to low monomer concentrations (0.2 M, 94% depolymerization to 2,3-DHB).^42^ Moreover, the enzymatic degradation of this homopolymer was facilitated by proteinase K, which led to a 39% decrease in molecular weight over 60 h. The *T*_g_ of PHEB was 30 °C, and it exhibited slow crystallization kinetics. Thermal degradation of PHEB occurs above 145 °C, limiting applications to low temperature.^43^ We tuned the thermal properties of PHEB by incorporating it into PLA.^44^ Copolymerization of 2,3-DHB with l-lactide increased the thermal properties and stability up to those of the triblock copolymer (*T*_g_ = 40 °C, *T*_m_ = 145 °C). Depolymerization does not occur without an accessible chain end, although selectively removing a single block in a block copolymer may facilitate sequential monomer isolation in chemical recycling systems.

**Figure 5:**
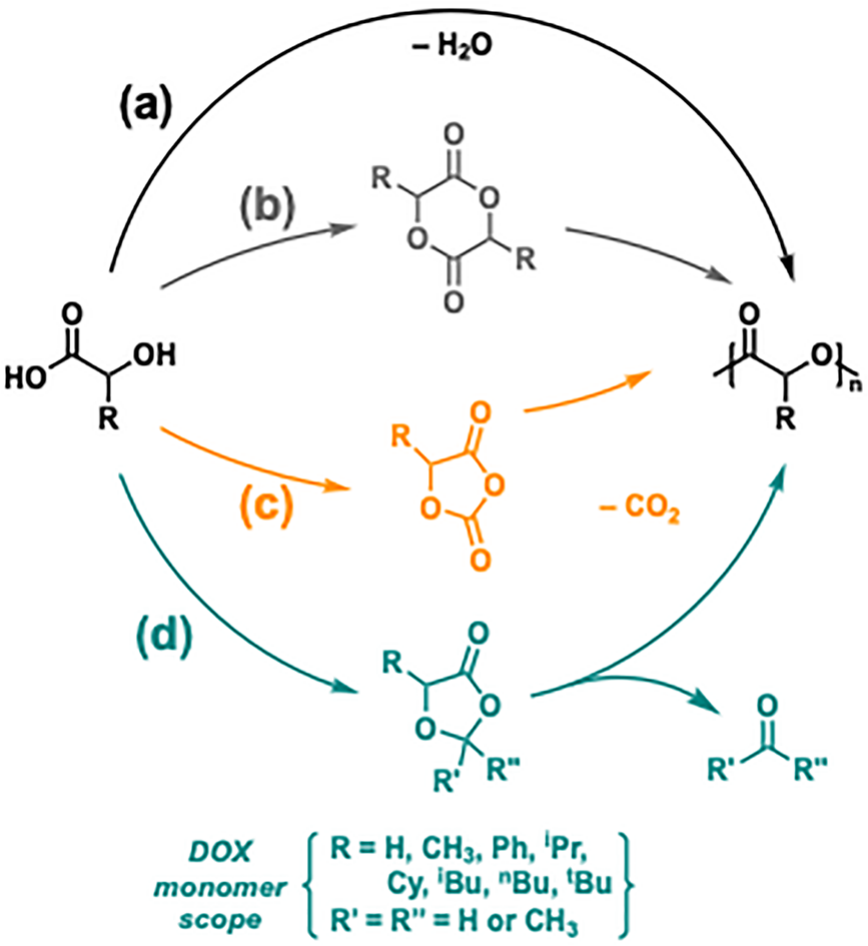
Synthesis of poly(α-hydroxy acid)s via (a) polycondensation or the ring-opening polymerization of (b) lactones, (c) *O-*carboxyanhydrides, and (d) 1,3-dioxolan-4-one (DOX) monomers.

For each of these polymeric targets, the products are somewhat esoteric and likely to be expensive. In our efforts to consider economic sustainability, we recently explored an underutilized synthetic strategy to expand the scope of conventional polyesters through the polymerization of 1,3-dioxolan-4-one (DOX) monomers. Our goal was to overcome key limitations in existing synthetic strategies: the polycondensation of α-hydroxy acids affords oligomeric and/or polydisperse products (Figure 5a), whereas introducing side-chain functionality into lactide derivatives is challenging and low-yielding (Figure 5b). To address this, Bourissou and co-workers developed *O*-carboxyanhydrides that enabled an array of functional poly(α-hydroxy acid)s (Figure 5c),^45^ although the associated monomer syntheses require phosgene or diphosgene and thus present some sustainability concerns. These new DOX monomers have a broad functional group tolerance, affording structurally divergent polyesters upon elimination of small molecules such as formaldehyde and acetone (Figure 5d), and are prepared from often-renewable α-hydroxy acids in high yields with atom economy.

**C18** catalyzes the ROP of MeDOX (R = CH_3_) to form isotactic PLA with a *T*_g_ of 59 °C and a *T*_m_ of 153 °C with quantitative acetal elimination. *i*-Propyl-, *n*-butyl-, *i*-butyl-, *t*-butyl-, and cyclohexyl-substituted polyester are all readily prepared. The rate of polymerization is tied to monomer sterics, as evidenced by the sluggish polymerization of *t*-butyl-substituted DOX.^2^ The *M*_n_ remained lower than expected because of a competing Al-catalyzed Tishchenko reaction, wherein formaldehyde and the propagating alkoxide species disproportionate to form the corresponding ester and methanol. The latter acts as a chain-transfer agent and reduces molecular weights significantly. Optimized conditions were developed, applying dynamic vacuum in jacketed, cooled ampules, ultimately achieving high molecular weights. In addition, catalyst **C26** strips bulk away from the Al center, increasing propagation rates compared to the Tishchenko reaction. Together these allow for isotactic poly(mandelic acid), a potential biodegradable alternative to polystyrene, to be prepared in high yield and good molecular weights (21–50 kg mol^–1^) from PhDOX.^46^

The same ring-opening elimination strategy also works for other monomers. The exocyclic poly(2-hydroxy-cyclopentane-1-carboxylic acid) bears cyclopentyl repeat units in the main chain and was synthesized through the ROP of a bicyclic cyclopentyl-1,3-dioxane-4-one. Catalyst screening showed ZnEt_2_ to be the preferred catalyst in homopolymerizations, albeit with low molecular weights, while **C18** and **C26** were preferred for copolymerizations with more traditional cyclic esters such as ε-caprolactone and *rac-*lactide. Interestingly, this dramatically increased both insertion rates, highlighting the importance of understanding reactivity ratios.^47^

This evolution of catalysts, monomers, and polymers may provide foundations for sustainable polymers, but in and of themselves, they are not sustainable. How can we ensure that our imagined fates for these materials are realized?

## Systems and Fates

The increasing awareness of global plastic consumption and waste has created a societal, industrial, and governmental impetus to change our plastics use to a more circular model. Plastics themselves are remarkably durable and versatile materials that often outperform alternatives for a fraction of the cost (and energy), leading to their prolific, widespread, and necessary use. When envisaging a more sustainable (circular) materials economy, we must therefore maintain a balanced view that includes the social and economic benefits of plastics. Equally important to this venture is an understanding of practice and infrastructure, both current and forecasted, because these define the real-world parameters that will realize, or worse, undermine, the success of new materials, technologies, and policies. With this backdrop, it becomes possible to facilitate positive change that is both sensible in the present moment yet adaptable to future, increasingly ambitious goals.

Evaluating material circularity requires an understanding of both production and end-of-life opportunities, which span from reuse to recycling to biodegradation. Life cycle analysis is an important tool that enables comparisons of these separate fates and the associated utilization of resources. However, the boundaries that are set during such analyses have an immense effect on their outcome, which is often a distinct challenge that requires cross-referencing with the real world. Furthermore, the potential for an end-of-life fate is no guarantee: a material can be “reusable”, “recyclable”, or “compostable”, but these attributes mean little if it is not actually reused, recycled, or composted. Thus, a shift to focus on past-tense terminology is a crucial adjustment that must be made by specialists, politicians, and the public at large. To evaluate whether a material is or can be circularized, a systems-level approach that elucidates the upstream and downstream realities is paramount. We have recently demonstrated such an approach that contextualizes household plastic waste in the U.K. and offers a potential improvement based on social practice and the regional complexities of U.K. waste management.^3^

**Figure 6:**
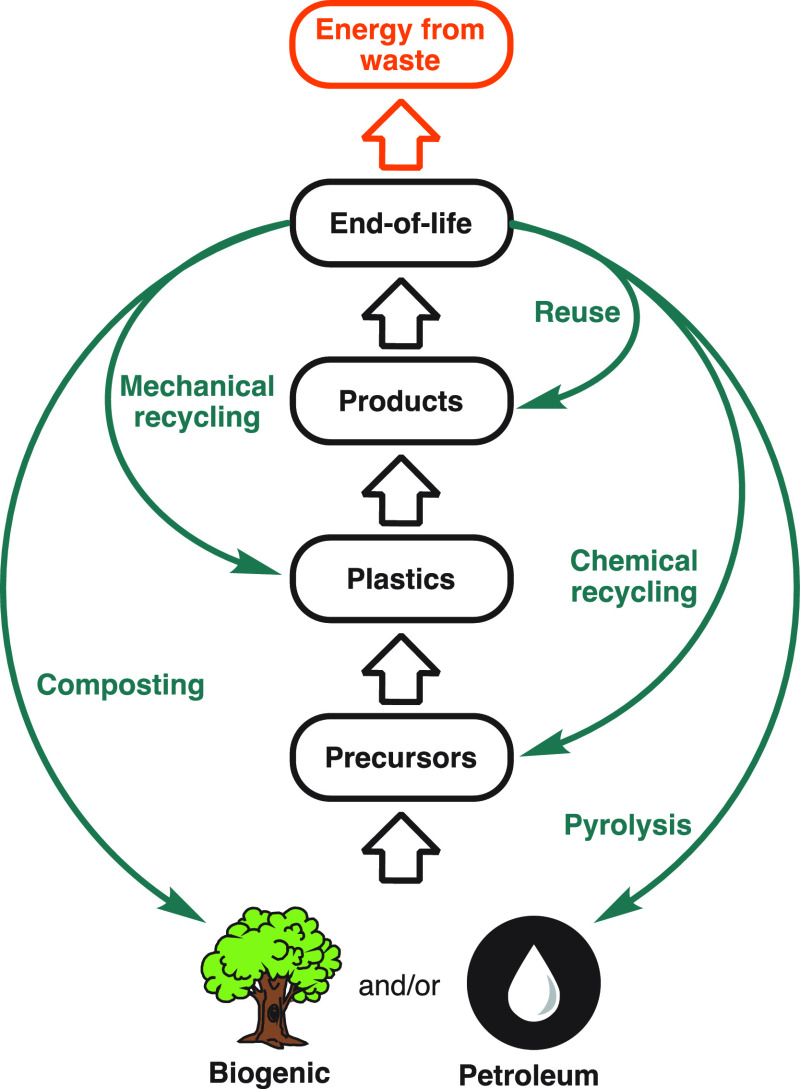
Potential end-of-life fates to enable a more circular plastics economy.

Armed with both systems-level knowledge and an understanding of end-of-life opportunities, we must seek to retain as much value from plastic waste as possible. Ideally, this should be accomplished while minimizing associated infrastructural costs (energy, transport, etc.), continually improving on and investing in existing infrastructure (energy from waste, composting, and mechanical recycling), and translating emerging technologies (pyrolysis and chemical recycling) to an industrial scale. In tandem, these aspects will enable more efficient and numerous circular loops in materials use, production, and disposal (Figure 6).

The above undertaking requires significant interaction and symbiosis among academia, industry, and policymakers. Industry must guide the way toward employing materials that are most amenable to closed loops. Industry and government must work together to develop programs that inform and incentivize collection, reuse, and recycling. For example, deposit return schemes could promote the reprocessing of various high-value plastics and the reuse of materials that have high energy costs associated with their reprocessing (e.g., glass and metal). Government needs to work with academia and industry to solve problems associated with circularizing plastic products, such as the current lack of consistent quality of plastic and the lack of relationships between waste collection infrastructure and production infrastructure (i.e., chemical manufacturers, virgin plastic producers, and plastics converters). Standardizing product design in various sectors (food, medical, consumer goods, etc.) is key to leveling the playing field across manufacturers and enabling more streamlined waste streams. Government and academia should work together to develop new approaches to the reliable quantification of recycled content in products as well as the evaluation of reuse versus recycling schemes for a target application. Finally, academia and industry should collaboratively ascertain which of the many potential fates is best suited for a given product.

A calculated push toward optimum reduction and reuse of plastics is key above all else. Reduction inherently limits plastic waste production, but it is important to strike the right balance and avoid wasting other materials, for example, food, fuel, and medical supplies. Products should be designed explicitly for function while minimizing material, unnecessary plastic complexity, and aesthetic additives (e.g., colorants). Furthermore, products should be developed with an increasing consideration of waste management; wherever possible, disparate components should be easy to disassemble and sort. Taken together, these criteria maintain the societal and economic value of the product while maximizing the potential for its material value to be recovered after its serviceable life. Depending on transport distances and cleaning protocols, reuse models may enable the most value to be recovered from a product. With such models, materials must be durable and trackable through multiple iterations of service. These additional constraints provoke the development of robust tagging technologies, plastic toughening strategies, and new resilient plastics.^48^

Mechanical recycling has a relatively low energy demand, can be implemented with existing industrial infrastructure, and directly facilitates the use of waste plastic to displace virgin plastic production. These attributes make it particularly appealing as a major target for sustainable systems in the short term. The main limitations are the need for clean feedstocks and the inevitable eventual degradation of any plastic over multiple extrusion cycles.^49^ Both are partially counteracted by the use of additives and feedstock supplementation using virgin plastic. Given the immediate feasibility of mechanical recycling and its potential to enable products to be circularized, it is imperative that research in this space is conducted on both a technical and systemic level. Cutting-edge technologies should focus on mitigating and reversing degradation through improved recycling additives, compatibilizers, and solid-state (re)polymerization methods.^50^ On a systems level, circularity will best be enabled with a synchronized effort from government, academia, and industry to facilitate the maximum recovery of usable plastic waste from both households and businesses. Inherent in this venture is the necessity to quantify and monitor the recycled content that is in any discarded plastic item; without this knowledge, the economics of the recycling industry will be less stable to fluctuations in feedstock quality. An overarching need for both technologies and systems is transparency and consistency in additives and formulations because this will promote efficacy across postconsumer recycling plants as well as enable the identification and suppression of ecotoxicological risk.^51,52^ This extends to potentially toxic catalyst residues, including both metals (Sn, Al, etc.) and organocatalysts.

A complementary way to valorize plastic waste is chemical recycling, which is emerging as a promising strategy to enable material circularity. Importantly, the products of chemical recycling can be the same precursors to the original product, potentially enabling “infinite” loops, or other value-added chemicals. Another attractive feature is the potential to recover usable feedstocks from nonseparable and/or nonreprocessable waste streams (e.g., multilayered materials). The key challenges that must be addressed to realize chemical recycling are process selectivity and the associated yields and energy costs. Research efforts should optimize either the selective recovery of pure chemicals from uniform waste (chemical recycling to monomers) or the efficient recovery of a broad mixture of usable chemicals from mixed-waste feedstocks (broad-scope pyrolysis). The former is well exemplified by recent developments in chemolysis and thermal depolymerization, which are especially promising for nonhydrocarbon polymers such as polyesters, polyamides, and polyacetals.^53,54^ Beyond pyrolysis, there are intriguing selective-dissolution processes emerging that may also be suited for mixed-waste or multilayered materials.^50^ In all cases, chemical recycling must be selective, fast, and amenable to real postconsumer waste.

Biodegradation, either in natural systems or under engineered composting conditions, has an important place in the sustainability landscape of the future.^55,56^ However, the predictability of biodegradation is a confluence of material properties and those of the receiving environment (e.g., terrestrial, marine, and compost). It is therefore difficult to ensure full biodegradation to carbon dioxide, methane, water, and biomass with no accumulation of toxic compounds. Insufficient biodegradation may instead yield the ready formation of microplastics or nanoplastics, the full ecotoxicological effects of which are yet unknown for both humans and wildlife.^57^ Beyond toxicity evaluations of plastic fragments and relevant chemicals (e.g., additives and catalyst residues), further research on structure–property–biodegradation relationships, especially across multiple receiving environments, is needed to substantiate the material design and claims of biodegradability. Finally, social practice with biodegradable products is a difficult hurdle because consumers may be more likely to litter or misplace the products in recycling or food waste. These caveats therefore suggest that circularity via biodegradation should be resorted to only if the application precludes material recovery and if significant proof of nondetrimental biodegradation exists. This should be contrasted with a focus on biobased strategies, which will likely be a focus of other contributions in this special issue, where the potential to sequester CO_2_ in long-life products holds important potential.

The future is uncertain, but our ambition should be clear. We must value plastic for what it is: a remarkably versatile, durable material whose benefits to us as a society are innumerable. Not using plastic in the future would be to our detriment, but using plastic as wastefully as we have grown to do is a foundation upon which we can and must improve. Plastic should be cherished as a valuable and even dangerous resource, calling for its proper management after use such that it may remain in circulation as long as possible before returning to raw chemicals or energy. This recovery of value promotes an opportunity for economic growth, less pollution, and a more efficient materials sector.

There is no single catalyst that suits every monomer, no monomer that suits every application of polymer science, and no end of life that suits all. We must recognize the nuances of the field in which we work, building sustainable collaborations that deliver the urgent plastics innovations the world needs.
